# Genetic Polymorphisms of *GP1BA*, *PEAR1,* and *PAI-1* may be Associated with Serum sIgE and Blood Eosinophil Levels in Chinese Patients with Allergic Diseases

**DOI:** 10.2174/0118715303285101240118062549

**Published:** 2024-01-30

**Authors:** Rui Tang, Xiaohong Lyu, Jinlyu Sun, Hong Li

**Affiliations:** 1 State Key Laboratory of Complex Severe and Rare Diseases, Allergy Department, Peking Union Medical College Hospital, Chinese Academy of Medical Sciences and Peking Union Medical College, Beijing, China;; 2 Beijing Key Laboratory of Precision Medicine for Diagnosis and Treatment of Allergic Diseases, Allergy Department, National Clinical Research Center for Dermatologic and Immunologic Diseases, Peking Union Medical College Hospital, Chinese Academy of Medical Sciences and Peking Union Medical College, Beijing, China;; 3 Eight-year Program of Clinical Medicine, Chinese Academy of Medical Sciences, Peking Union Medical College, Beijing, China

**Keywords:** Allergy and immunology, gene polymorphism, platelet endothelial aggregation receptor 1 (PEAR1), specific immunoglobulin E (sIgE), eosinophil, glycoprotein Ib alpha gene (GP1BA), plasminogen activator inhibitor 1 gene (PAI-1)

## Abstract

**Background:**

It has been suggested that genetic factors may be substantially linked to allergy disorders.

**Objective:**

This study aims to investigate the relationship between the serum specific Immunoglobulin E (sIgE), blood eosinophil, and the polymorphisms of glycoprotein Ib alpha gene (*GP1BA*) rs6065, platelet endothelial aggregation receptor 1 gene (*PEAR1*) rs12041331, and plasminogen activator inhibitor 1 gene (*PAI-1*) rs1799762.

**Methods:**

From the Peking Union Medical College Hospital, this study enrolled 60 healthy participants and 283 participants with allergic diseases. TaqMan-minor groove binder (MGB) quantitative polymerase chain reaction (qPCR) was used to examine the gene polymorphisms in each group.

**Results:**

The TaqMan-MGB qPCR results were completely consistent with the DNA sequencing results, according to other studies in this medical center (Kappa =1, *p* <0.001). The *GP1BA* rs6065, *PEAR1* rs12041331, and *PAI-1* rs1799762 polymorphisms did not show different distribution between allergy patients and healthy individuals. Concerning allergy patients, the CT (n=33) genotype of *GP1BA* rs6065 had higher blood eosinophil level than the CC (n=250) genotype (0.59, IQR 0.32-0.72 *vs* 0.31, IQR 0.15-0.61, *10^9^/L, *p* =0.005). The serum sIgE of AA (n=46) genotype of *PEAR1* rs12041331 was lower (median 3.7, interquartile quartiles (IQR) 0.2-16.8, kU/L) than the GA (n=136) and GG (n=101) genotypes (GA median 16.3, IQR 3.1-46.3, kU/L, *p* = 0.002; GG median 12.9, IQR 3.0-46.9, kU/L, *p* =0.003). The GA genotypes of *PEAR1* rs12041331were with higher blood eosinophil levels (median 0.42, IQR 0.17-0.74 *10^9^/L) than the AA genotype (median 0.25, IQR 0.15-0.41*10^9^/L, *p* =0.012). The sIgE of the 5G5G (n=44) genotype of *PAI-1* rs1799762 was lower (median 5.0, IQR 0.1-22.8, kU/L) than the 4G5G (n=144) (median 17.3, IQR 3.7-46.0, kU/L, *p* = 0.012).

**Conclusion:**

The *GP1BA* rs6065, *PEAR1* rs12041331, and *PAI-1* rs1799762 polymorphisms may be associated with the genetic susceptibility of serum sIgE or blood eosinophil in Chinese allergic disease patients.

## 1 INTRODUCTION

Allergic disorders have become a considerable social and economic burden due to their increasing prevalence [1-4]. The enormous population of China has made the prevalence of allergic diseases one of the major issues faced by the healthcare system of the country [5, 6]. Asthma, allergic and nonallergic rhinitis, and food allergies can all be diagnosed and treated through fundamental and clinical immunology research based on molecular and cellular mechanisms [7-10]. The serum-specific Immunoglobulin E (sIgE) and blood eosinophil levels play a crucial role in the diagnosis of allergic diseases [11-13].

Research has shown that inheritance plays a role in the development of allergies and related illnesses [14-16]. Genome-wide association studies (GWAS) have identified disease-related single nucleotide polymorphisms (SNPs) that can influence genetic susceptibility to allergic diseases [17-22]. Studies of monogenic disorders have also highlighted important cellular processes and protein functions involved in allergies [23-25]. Identifying crucial SNPs is important for understanding the development of allergic conditions. Serum sIgE and blood eosinophils are crucial biomarkers for the diagnosis of allergic diseases [26, 27]. Elevated levels of sIgE are commonly observed in patients with allergies and can provide important information about the allergen sensitivities of an individual [28]. Blood eosinophil counts are also elevated in many allergic conditions and can be used to monitor disease severity and response to treatment [29]. Together, these biomarkers play a critical role in the diagnosis and management of allergic diseases, allowing for more accurate and personalized treatment strategies. Previous studies have investigated the relationship between SNP genotypes and serum IgE [30-32] or blood eosinophils [33, 34].

In addition to the important role of serum sIgE and blood eosinophils in the diagnosis and management of allergic diseases, numerous potential genes have been identified as pathophysiological mediators involved in various allergic illnesses. For example, the glycoprotein Ib alpha gene (GP1BA) has been linked to severe, treatment-refractory asthma [35], while platelet endothelial aggregation receptor 1 (PEAR1) has been found to be highly correlated with cardiovascular illness. The unique relationship between Immunoglobulin E (IgE)-mediated allergy and cardiovascular disease was discovered to be PEAR1 [36]. Recent research has also highlighted the significant role of plasminogen activator inhibitor 1 (PAI-1) in controlling airway remodeling, hyperresponsiveness, and allergic inflammation, which may contribute to the onset of asthma [37-40]. Understanding the relationships between these genes and allergic diseases can lead to more personalized and effective treatment strategies. Previous studies have investigated the relationship between SNP genotypes and serum IgE or blood eosinophil, providing valuable insights into the gene polymorphisms to biomarkers.

The objective of this study is to investigate the potential association between gene polymorphisms in *GP1BA* rs6065, *PEAR1* rs12041331, and *PAI-1* rs1799762 and the levels of serum specific-IgE (sIgE) and blood eosinophils in Chinese patients with allergies.

## 2 MATERIALS AND METHODS

### 2.1 Ethics

This study involving human participants adhered to the ethical standards outlined in the 1964 Helsinki Declaration and its subsequent amendments, as determined by the institutional and/or national research committee. Approval for this study was obtained from the Chinese Academy of Medical Sciences and Peking Union Medical College Hospital Drug Clinical Trial Ethics Committee, with registration information No.002062 and ethics approval No. KS2019282.

### 2.2 Study Design and Participants

This prospective study was conducted at Peking Union Medical College Hospital (PUMCH) in Beijing, China, from July to December 2019. Patients underwent standard diagnostic workups based on their symptoms and auxiliary test results. Inclusion criteria for allergy patients were: [1] clinical diagnosis of allergy diseases, such as allergic rhinitis, asthma, urticaria, atopic dermatitis, cough, atopic conjunctivitis, eczema, or a history of severe anaphylactic reaction; [2] positive results for serum specific IgE, skin prick test, or intradermal test. Exclusion criteria for allergy patients were: [1] the presence of serious comorbidities, such as diabetes, liver disease, kidney disease, and [2] immunocompromised status.

The inclusion criteria for healthy participants were: [1] absence of any symptoms related to allergic diseases, including allergic rhinitis, allergic asthma, atopic dermatitis, allergic conjunctivitis, *etc*.; [2] no personal or family history of allergic diseases; [3] no other immune system diseases; [4] no organic diseases; [5] voluntary participation in disease-related questionnaires; and [6] no participation in any drug clinical trials within 3 months. The exclusion criteria for healthy participants were: [1] history of allergic diseases or chronic medical conditions associated with allergy diseases in this study; [2] history of significant allergen exposure; [3] presence of serious comorbidities, such as diabetes, liver disease, kidney disease, *etc*.; [4] and immunocompromised status.

### 2.3 Clinical Information Collecting

After informed consent, we collected information on the plasma allergen sIgE levels, disease duration (years), and blood eosinophil counts of the patients, and whether desensitization was used. The measurement of serum sIgE levels was taken as the highest value of serum sIgE for various common allergens, including pollen, dust mites, molds, cat hair, *etc*. The demographic information about the participants of the study is displayed in Table **1**.

### 2.4 TaqMan-MGB qPCR Method

We extracted genomic DNA from peripheral blood samples using DNA extraction kits from Tianlong Technology Co. LTD in Xi'an, China. The GP1BA rs6065, PEAR1 rs12041331, and PAI-1 rs1799762 genes were genotyped using a gene polymorphisms RT-PCR detection kit from Wuhan HealthCare Biotechnology Co., Ltd., Wuhan, China. TaqMan chemistry was used to genotype the genes according to the Applied Biosystems methodology with real-time Prism 3730XL Sequence Detection System (ABI Inc. CA, United States). In our previous studies, we demonstrated the effectiveness of the TaqMan-MGB qPCR kit for detecting gene polymorphisms [41-44]. To examine the agreement between DNA sequencing and TaqMan-MGB qPCR, we used the Kappa test with a Kappa value of 1 and a *p* value < 0.001.

### 2.5 Data Statistics and Analysis

We used SPSS 26.0 (SPSS Inc., Chicago, IL, United States), R Project (version 4.2.0), and RStudio (Open-Source Edition) software to analyze the data. The Hardy-Weinberg equilibrium (HWE) test was used to assess whether the frequency distribution of polymorphisms across genomes was representative. The Wilcoxon or Chi-square test was used to determine whether there was a significant difference in SNP between the illness group and the healthy control group. Non-parametric tests, such as the Mann-Whitney U test and the Kruskal-Wallis test, were utilized to analyze differences in clinical indicators between allergy patients with different genotypes. Statistical differences were considered significant at *p* <0.05, except for the two-by-two comparisons between the three groups of patients, where statistical differences were considered significant at *p* <0.025.

## 3 RESULTS

### 3.1 Demographic Characteristics of Participants

Table **1** displays the demographic information of the study participants, which consisted of 283 patients with allergic diseases and 60 healthy adults as controls. The median age of the patients was 29, with 129 men and 154 women. Based on clinical performance, there were 276 patients with allergic rhinitis, 62 with asthma, 34 with atopic conjunctivitis, and 18 with cough. The median duration of allergic diseases for all patients was 4 (IQR, 2-7) years, with a median serum allergen-sIgE of 12.6 (IQR, 2.2-42.8) kU/L and a median blood eosinophil count of 0.33 (IQR, 0.17-0.64) *10^9^/L. Desensitization treatment was administered to 17.7% of allergy patients.

Fig. (**1**) displays the amplification plots for the genotypes of *GP1BA* rs6065, *PEAR1* rs12041331, and *PAI-1* rs1799762. The frequency distributions of these polymorphisms in allergy patients followed the Hardy-Weinberg equilibrium law (*p* >0.05). Furthermore, the genotype distribution of all allergic patients in these three genes showed no significant difference compared to that of healthy individuals, as indicated in Table **1**.

### 3.2 Analysis of Association of *GP1BA* rs6065 with Serum sIgE and Eosinophils in Allergy Patient

**Fig. (2):**
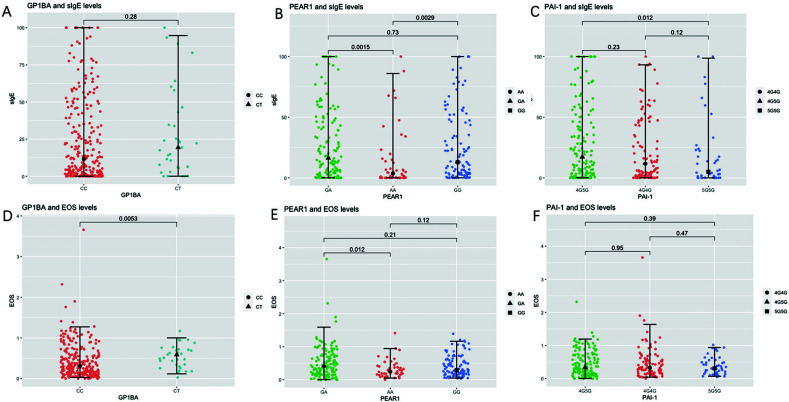
(**A**-**C**) Scatter plots and two-by-two comparisons of different genotypes of *GP1BA* rs6065, *PEAR1* rs12041331, *PAI-1* rs1799762 against serum sIgE. (**D**-**F)** Scatter plots of different genotypes of the three genes *versus* blood eosinophil levels and two-by-two comparisons. Note: Black dots and black lines inside the scatterplot represent medians and interquartile quartiles. The numbers on the horizontal line at the top of the picture represent *p*-values for two-by-two comparisons. In Figs. (**A** and **D**), *p* <0.05 is considered statistically different; in Figs. (**B**, **C**, **E**, and **F**), *p* <0.025 is considered statistically different.

Table **2** presents a comparison of age, gender, disease duration, serum sIgE, and blood eosinophil levels in allergic patients with different genotypes of *GP1BA* rs6065 (C5792T). The CT genotype (n=33) of *GP1BA* rs6065 was associated with higher blood eosinophil levels compared to the CC genotype (n=250) (0.59, IQR 0.32-0.72 *vs* 0.31, IQR 0.15-0.61, *10^9^/L, *p* =0.005) (Table **2** and Fig. **2A**-**D**). However, no significant differences were observed between the two genotypes for the other variables analyzed.

### 3.3 Analysis of Association of *PEAR1* rs12041331 with Serum sIgE and Eosinophils in Allergy Patient

The Kruskal-Wallis test revealed that the serum sIgE levels of allergy patients with AA (n=46), GA (n=136), and GG (n=101) genotypes of *PEAR1* rs12041331 were statistically different (*p* =0.004, Table **3**). Specifically, the AA genotype was associated with lower serum sIgE levels (median 3.7, IQR 0.2-16.8, kU/L) compared to the GA genotypes (median 16.3, IQR 3.1-46.3, kU/L, *P*=0.002) and the GG genotypes (median 12.9, IQR 3.0-46.9, kU/L, *p* = 0.003) (Fig. **2B**).

Additionally, the blood eosinophil levels were also found to be statistically different among AA, GA, and GG genotypes of PEAR1 rs12041331 (*p* =0.036, Table **3**). Specifically, the GA genotypes were associated with higher blood eosinophil levels (median 0.42, IQR 0.17-0.74 109/L) compared to the AA genotype (median 0.25, IQR 0.15-0.41, 10^9^/L, *p* =0.012, Fig. **2E** and **F**). However, no statistically significant differences were observed in age, gender, or duration of disease among allergic patients with different genotypes in the *PEAR1* rs12041331 gene (Table **3**).

### 3.4 Analysis of Association of *PAI-1* rs1799762 with Serum sIgE and Eosinophils in Allergy Patient

According to the Kruskal-Wallis test, the serum sIgE levels of allergy patients with 4G4G (n=95), 4G5G (n=144), and 5G5G (n=44) genotypes of *PAI-1* rs1799762 (-675,4G5G) were found to be statistically different (*p* =0.036, Table **4**). A two-by-two comparison revealed that the serum sIgE levels of patients with the 5G5G genotype (n=44) of *PAI-1* rs1799762 were lower (median 5.0, IQR 0.1-22.8, kU/L) compared to those with the 4G5G genotype (n=144) (median 17.3, IQR 3.7-46.0, kU/L, *p* = 0.012) (Fig. **2C**). No statistically significant differences were observed in age, gender, duration of disease, or blood eosinophil levels among allergic patients with different genotypes in the *PAI-1* rs1799762 (-675,4G5G) (Table **4**).

## 4 DISCUSSION

Allergic disorders pose a significant health risk to patients and can even be fatal in some cases [45, 46]. The identification of SNPs that are associated with disease risk and prognosis is crucial for personalized medicine. Despite the growing body of research in this area, there have been limited studies investigating the role of SNPs in allergic diseases. The TaqMan-MGB qPCR method has emerged as a reliable and cost-effective approach for investigating SNPs in patients with allergy disorders. Using this technology, we were able to identify that SNP may have an impact on serum sIgE levels and blood eosinophil counts, which are important indicators of allergic inflammation. Further investigations into the role of SNPs in allergic diseases could provide valuable insights into disease mechanisms and personalized treatment options.

This study provides evidence of a potential correlation between genotypes of *GP1BA* rs6065, *PEAR1* rs12041331, and *PAI-1* rs1799762 and serum sIgE levels and blood eosinophil counts in Chinese allergy patients. GP1BA rs6065 has been previously reported in severe anaphylaxis patients, where it was found to be significantly downregulated compared to healthy controls [47]. However, the underlying mechanisms of this downregulation remain poorly understood. In this study, we found that the CT genotype of *GP1BA* rs6065 was associated with higher blood eosinophil levels compared to the CC genotype in allergy patients. These findings may provide a theoretical basis for further exploration of the impact of *GP1BA* polymorphisms on allergic reactions.

Furthermore, our study highlights the potential significance of *PEAR1* rs12041331 in the pathogenesis of allergic diseases. Previous research has identified the high-affinity IgE receptor subunit α [FcεR1α] as a ligand for PEAR1 [36]. Oligomerized FcεR1α has been shown to enhance platelet aggregation and induce phosphorylation of PEAR1. Interestingly, this effect is inhibited by IgE. In our study, we observed a correlation between the genotypes of *PEAR1* rs12041331 and serum sIgE levels. Specifically, individuals with the AA genotype had lower serum sIgE levels compared to those with the GA and GG genotypes. Additionally, we found an association between the GA genotypes of *PEAR1* rs12041331 and higher blood eosinophil levels compared to the AA genotype. These findings provide further evidence of the potential role of *PEAR1* rs12041331 in modulating allergic responses and highlight its involvement in the regulation of both serum sIgE levels and blood eosinophil counts.

PAI-1, a key inhibitor of the fibrinolytic system, has been implicated in various disorders, including thrombosis, systemic lupus erythematosus, thyroid eye disease, and metabolic syndrome. Recent research indicates that PAI-1 also plays a role in controlling airway remodeling, hyperresponsiveness, and allergic inflammation, which are associated with the development of asthma [37]. Concerning asthma, studies have investigated the association between *PAI-1* 4G/5G polymorphisms and the risk of IgE-mediated asthma and allergy disorders. A meta-analysis conducted in 2012, involving 1817 cases and 2327 controls, identified the -675 4G/5G polymorphism of the *PAI-1* gene as a potential risk factor for asthma [48]. Previous findings revealed a significant association between the *PAI-1* promoter polymorphism and IgE-mediated allergic diseases as a whole [49]. Animal experiments [50] have shown that the double knockout of α2-antiplasmin and *PAI-1* genes leads to an increase in plasma IgE levels with age, exceeding 1000 ng/mL after 6 months. The plasma cells producing IgE were detected in perivascular assembled lymphocytes.

In our previous retrospective study of the same cohort, we found that the distribution of PAI-1 rs1799762 was different between patients with allergic coughs and healthy people. Concerning cough patients, the 4G4G and 5G5G genotypes of PAI-1 rs1799762 were more frequent than healthy people. In the previous study, we carefully discussed the association between different genotypes and symptoms in allergy patients but left out the analysis between genotypes and blood indicators. There is still a research gap between the two. In this new research, we observed that individuals with the 5G5G genotype of PAI-1 rs1799762 had lower levels of sIgE compared to those with the 4G5G genotype. Our two studies were only observationally analyzed from a cross-sectional study, and more in-depth causal analyses may be needed in the future to explore the association between phenotype and sIgE levels in patients with different genotypes.

Based on the results of this study, it can be concluded that the *GP1BA* rs6065, *PEAR1* rs12041331, and *PAI-1* rs1799762 polymorphisms may be associated with the genetic susceptibility of serum sIgE or blood eosinophil in Chinese allergic disease patients. These findings could have clinical implications for the diagnosis and treatment of allergic diseases. For instance, patients with the CT genotype of *GP1BA* rs6065 or GA genotype of *PEAR1* rs12041331 may have a higher blood eosinophil level and may benefit from more precise treatment [51]. Additionally, patients with the AA genotype of *PEAR1* rs12041331 or 5G5G genotype of *PAI-1* rs1799762 may have a lower serum sIgE level, which could be taken into consideration when interpreting allergy test results. Further studies are needed to validate these findings and to explore their potential clinical applications.

Despite the positive findings of our study, several limitations must be acknowledged. The small sample size of our study might have affected the statistical power of our analysis, particularly for some subgroups. Therefore, future multi-center, large-scale, longitudinal studies are needed to confirm and expand upon our findings regarding the polymorphisms and allergic biomarkers in allergic patients. Additionally, we did not investigate the relationship between the three gene polymorphisms and their levels in allergic individuals, which warrants further investigation for the development of prognostic factors and therapeutic targets for allergic disorders. Despite these limitations, our study provides valuable insights into the study of multiple loci polymorphisms in allergic biomarkers, which highlights the need for further research in this field.

## CONCLUSION

In conclusion, our study found that the *GP1BA* rs6065, *PEAR1* rs12041331, and *PAI-1* rs1799762 polymorphisms may be associated with the genetic susceptibility of serum sIgE or blood eosinophil in Chinese allergic disease patients. Specifically, the CT genotype of *GP1BA* rs6065 was associated with higher blood eosinophil levels, while the AA genotype of *PEAR1* rs12041331 was associated with lower serum sIgE levels. Conversely, the GA genotype of *PEAR1* rs12041331 was associated with higher blood eosinophil levels. Additionally, the 5G5G genotype of *PAI-1* rs1799762 was associated with lower sIgE levels compared to the 4G5G genotype. These findings suggest that genetic variations in these polymorphisms may play a role in the development and progression of allergic diseases in the Chinese population.

## Figures and Tables

**Fig. (1):**
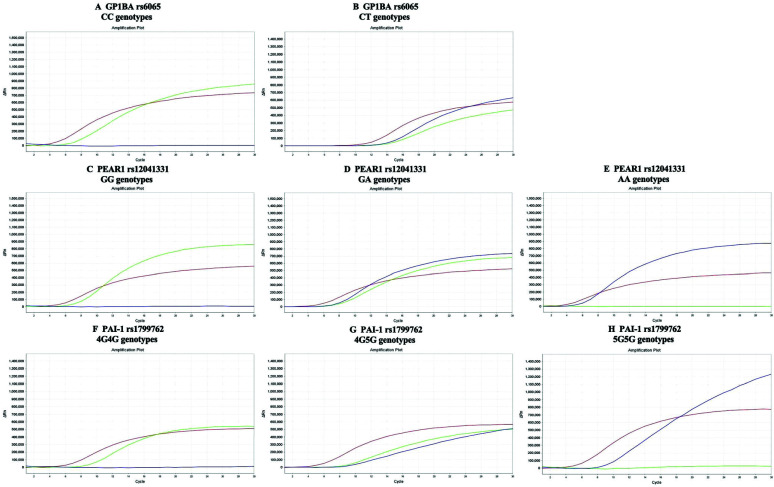
Amplification plots for genotypes of *GP1BA* rs6065, *PEAR1* rs12041331, and *PAI-1* rs1799762. (**A** and **B**) amplification plots of *GP1BA* rs6065 CC and CT genotypes. (**C**-**E**) amplification plots of *PEAR1* rs12041331 GG, GA and AA genotypes. (**F**-**H**) amplification plots of *PAI-1* rs1799762 4G4G, 4G5G and 5G5G genotypes.

**Table 1:** The demographic characteristics of the participants in this study.

-		**All Allergic Disease Subjects (n=283)**	**Healthy Individuals (n=60)**	***p*-value**
**Age (years, median, IQR)**		29 (13,40)	34 (31, 39)	0.700
**Gender-male, n (%)**		129 (45.6)	12 (20.0)	<0.001
**Duration of disease (years, median, IQR)**		4(2,7)	-	
**The allergen sIgE value (kU/L, median, IQR)**		12.6 (2.2, 42.8)	-	
Eosinophils counts (*10^9^/L, median, IQR)		0.33 (0.17, 0.64)	-	
**Desensitization treatment, n (%)**		50 (17.7)	-	
**Allergy symptoms, n (%)**	**Allergic rhinitis**	276 (97.5)	-	
	**Asthma**	62 (21.9)	-	
	**Atopic conjunctivitis**	34 (12.0)	-	
	**Cough**	18 (6.4)	-	
**Allergens corresponding to the highest sIgE values, n (%)**	**Dermatophagoides farina**	40 (14.1)	-	
	**Dermatophagoides pteronyssinus**	24 (8.5)	-	
	**Artemisia**	119 (42.0)	-	
	**Ragweed**	16 (5.7)	-	
***GP1BA* rs6065 (C5792T), n (%)**	**CC**	250 (88.3)	54 (90.0)	0.8853
	**CT**	33 (11.7)	6 (10.0)	
***PEAR1* rs12041331 (G2266A), n (%)**	**AA**	46 (16.3)	8 (13.3)	0.7307
	**GA**	136 (48.1)	32 (53.3)	
	**GG**	101(35.7)	20 (33.3)	
***PAI-1* rs1799762 (−675,4G5G), n (%)**	**4G4G**	95 (33.6)	16 (26.7)	0.4332
	**4G5G**	144 (50.9)	36 (60.0)	
	**5G5G**	44 (15.5)	8 (13.3)	

**Table 2:** Analysis of association of *GP1BA* rs6065 (C5792T) with genetic susceptibility to clinical characteristics.

**Genotypes**	**CC (n=250)**	**CT (n=33)**	***p*-value**
Age (years, median, IQR)	29 (13, 40)	30 (11, 38)	0.934
Gender-male, n (%)	114 (45.6)	15 (45.5)	0.987
Duration of disease (years, median, IQR)	4 (2, 7)	4 (1, 7)	0.681
The allergen sIgE value (kU/L, median, IQR)	11.7 (1.9, 42.8)	19.4 (5.8, 44.8)	0.275
Eosinophils counts (*10^9^/L, median, IQR)	0.31 (0.15, 0.61)	0.59 (0.32, 0.72)	0.005

**Table 3:** Analysis of Association of *PEAR1* rs12041331 (G2266A) with genetic susceptibility to clinical characteristics.

**Genotypes**	**AA (n=46)**	**GA (n=136)**	**GG (n=101)**	***p*-value**
Age (years, median, IQR)	29(9,43)	30(12,39)	30(14,39)	0.642
Gender-male, n (%)	20(43.5)	70(51.5)	39(38.6)	0.138
Duration of disease (years, median, IQR)	3(1,7)	4(2,7)	3(2,7)	0.642
The allergen sIgE value (kU/L, median, IQR)	3.7(0.2,16.8)	16.3(3.1,46.3)	12.9(3.0,46.9)	0.004
Eosinophils counts (*10^9^/L, median, IQR)	0.25(0.15,0.41)	0.42(0.17,0.74)	0.29(0.17,0.61)	0.036

**Table 4:** Analysis of association of *PAI-1* rs1799762 (-675,4G5G) with genetic susceptibility to clinical characteristics.

**Genotypes**	**4G4G (n=95)**	**4G5G (n=144)**	**5G5G (n=44)**	***p*-value**
Age (years, median, IQR)	28(12,41)	30(13,40)	31(16,39)	0.870
Gender-male, n (%)	46(48.4)	67(46.5)	16(36.4)	0.393
Duration of disease (years, median, IQR)	4(1,9)	4(2,7)	4(2,6)	0.896
The allergen sIgE value (kU/L, median, IQR)	11.6(1.9,46.4)	17.3(3.7,46.0)	5.0(0.1,22.8)	0.036
Eosinophils counts (*10^9^/L, median, IQR)	0.33(0.18,0.61)	0.34(0.16,0.72)	0.31(0.14,0.54)	0.682

## Data Availability

The data and supportive information are available within the article.

## LIST OF ABBREVIATIONS

GWAS: Genome-wide Association Studies
PUMCH: Peking Union Medical College Hospital
SNPs: Single Nucleotide Polymorphisms

## STANDARDS OF REPORTING

STROBE guidelines were followed.
